# EEG Feature Extraction and Data Augmentation in Emotion Recognition

**DOI:** 10.1155/2022/7028517

**Published:** 2022-03-28

**Authors:** Mahsa Pourhosein Kalashami, Mir Mohsen Pedram, Hossein Sadr

**Affiliations:** ^1^Department of Electrical and Computer Engineering, Faculty of Engineering, Kharazmi University, Tehran 15719-14911, Iran; ^2^Department of Computer Engineering, Rahbord Shomal Institute of Higher Education, Rasht, Iran

## Abstract

Emotion recognition is a challenging problem in Brain-Computer Interaction (BCI). Electroencephalogram (EEG) gives unique information about brain activities that are created due to emotional stimuli. This is one of the most substantial advantages of brain signals in comparison to facial expression, tone of voice, or speech in emotion recognition tasks. However, the lack of EEG data and high dimensional EEG recordings lead to difficulties in building effective classifiers with high accuracy. In this study, data augmentation and feature extraction techniques are proposed to solve the lack of data problem and high dimensionality of data, respectively. In this study, the proposed method is based on deep generative models and a data augmentation strategy called Conditional Wasserstein GAN (CWGAN), which is applied to the extracted features to regenerate additional EEG features. DEAP dataset is used to evaluate the effectiveness of the proposed method. Finally, a standard support vector machine and a deep neural network with different tunes were implemented to build effective models. Experimental results show that using the additional augmented data enhances the performance of EEG-based emotion recognition models. Furthermore, the mean accuracy of classification after data augmentation is increased 6.5% for valence and 3.0% for arousal, respectively.

## 1. Introduction

These days, emotion recognition based on EEG signals using machine learning and deep learning has become very debatable in different fields of study. The EEG data generated in response to an emotional stimulus, compared with visual or speech signals, are unique and cannot be hidden by individuals, even when they try not to show their emotions. Additionally, neuroscientists are trying to find patterns of brain activities for different states of emotions and determine if these patterns are common among different people. Experimental results have shown there are neural signatures for three emotions: positive, neutral, and negative [1]. Feature engineering as a way of pattern recognition is another controversial issue that should be considered carefully in training a model. So how to extract meaningful brain activities from apparently meaningless and complex brain electrical signals is a big challenge for BCI [2]. Many methods have been proposed to improve performance in many aspects, including preprocessing, feature extraction, feature selection, and classification [3, 4].

Many EEG-based emotion recognition methods have been studied in recent years. The main focus of emotion recognition is on feature extraction and classification. Classifiers use features as input to identify the emotional states. There are various methods for feature extraction, such as the traditional method of feature engineering based on many signal processing techniques and statistics, or automatic feature engineering, which can be directly extracted by neural networks. Many studies have been done on both traditional and automatic feature engineering to propose an effective EEG-based emotion classification. Extracted features are given as input to effective standard machine learning models like SVM [5–10], KNN [10–12], etc. Lately, deep learning networks have shown significant power in feature extraction and classification tasks, and many researchers have applied different neural networks to EEG data [13–16] to enhance accuracy.

The lack of EEG training datasets, compared with visual and audio datasets, is still one of the primary challenges in EEG-based emotion recognition tasks based on deep learning models. There are only a few public datasets for EEG-based emotion recognition: SEED, DEAP, DREAMER, MAHNOB-HCI3, and MPED [14]. In addition, the scale of these datasets is much smaller than image datasets like ImageNet. A machine learning model would be more accurate if it could access more training data. Generating fake EEG data is a common solution to solve the lack of data problem. This method is called augmentation. Lately, a variety of different techniques have been used to generate more data. For example, applying a geometric modification to original data is commonly used for image data augmentation. In EEG data augmentation, Gaussian noise is usually added to data to create new data [13], but recently a new method has been proposed to generate EEG realistic-like data by using deep generative neural networks [17]. A CWGAN network is proposed in [17] for the first time to generate a vector of EEG features. Then, a technique is used to check the quality of generated data and only high-quality data are added to the trainset. Finally, SVM and DNN are trained to classify the original and augmented training data with binary classification. A 2-dimensional Arousal-Valence model is used to identify emotions from complex and nonstationary EEG data. The experimental results have shown that data augmentation improved the accuracy of classifiers.

The rest of the paper is organized as follows: Section 2 provides an overview of related work on generative and data augmentation methods for EEG-based emotion recognition. In Section 3, the implementation of the proposed method is discussed in detail.  Section 4 describes the DEAP datasets and presents the details of our experimental settings. The experimental results and comparison of the proposed method with different methods are presented in section 5. Finally, in section 6, we present the conclusions of our work.

## 2. Related Work

Due to the high costs and challenges of EEG data collection, most EEG public datasets are small and the number of recorded data from different participants is limited. This has a great impact on the accuracy of implemented machine learning models for prediction and classification tasks and imposes a huge challenge in EEG data classification. Working on a method to generate EEG fake data like real data, is a controversial issue to solve the lack of data problem in EEG-based emotion recognition tasks. In this paper, the performance of emotion recognition models with the standard machine learning models and deep neural networks are compared before and after data augmentation to check whether data augmentation was effective or not. The experimental results indicate that data augmentation method effectively improved the performance of models in some cases.

Data augmentation for EEG-based emotion recognition by adding Gaussian noise to the trainset is used in [13, 18]. New data augmentation with deep generative models is proposed to generate EEG fake data for the first time in [17], and the results have shown improvement in accuracy. The combination of three datasets, DEAP, DREAMER, and a dataset that they collected themselves, is used to solve the lack of data problem in emotion recognition tasks in [19]. In the last few years, much research has been conducted by deploying machine learning techniques to analyze EEG data for emotion recognition. SVM classifier is proposed as a classification model for the prediction of three emotional states and EEG time-frequency features are used as input data for implemented classifier [20]. In [21], authors have used KNN as a classifier and amplitude of the signal as input features to predict eight emotional states.

LSTM network is developed to recognize emotions from EEG data and raw EEG signals of the DEAP dataset are given to the network as input features. Feature extraction is done automatically by the LSTM network and a dense layer is used for classification. The average accuracy of implemented network for arousal, valence, and liking is 85.65%, 85.45%, and 87.99% respectively. The proposed method reached a high average accuracy in comparison with the traditional techniques [15]. A multilayer group classification model based on a stacked autoencoder (MESAE) has been proposed to identify emotions. On the DEAP dataset [22], the average accuracy of the model for binary prediction of excitement and valence parameters was 77%, 76%, and F-score 69% and 72%, respectively. Two convolutional neural networks with new architectures are proposed for biometric identification based on EEG signals in [23]. An ensemble deep neural network is proposed to explore the correlation between channels and contextual information of recorded data from EEG frames. The hybrid method is a combination of CNN and RNN networks [24]. A deep neural network has been proposed to detect emotions from EEG signals using the DEAP dataset. Two types of neural network architecture have been studied in this research: CNN and DNN. Both models are highly effective in categorizing user emotions when training on preprocessed data [1]. GELM model has been used to identify stable patterns over time and evaluate the stability of the emotion recognition model. Feature selection and classification of patterns of emotions are evaluated in the SEED and DEAP datasets [25]. A CWGAN network is proposed as a data augmentation technique to generate EEG data in the emotion recognition task. The mean classification accuracy based on the 2d-arousal-valence model on SVM and DNN for the DEAP dataset is 48.9% and 47.5% respectively [17]. A combination of three datasets, DEAP, DREAMER, and a proprietary data set that they collected themselves, is proposed in [19] to solve the lack of data problem in emotion recognition tasks. The total dataset is related to 60 participants, which is the largest number compared with other datasets. The accuracy of this method for valence and arousal is 70.26% and 72.42%, respectively. As mentioned above, the study of EEG-based emotional recognition has never stopped. Although many deep learning methods have been developed to identify emotions from EEG signals, proposing a suitable method is still in its infancy. Due to the limitation of EEG data collection, the labeled EEG samples that can be used for deep learning techniques for EEG-based emotional recognition is a significant challenge, and proposing a solution is still an issue.

## 3. DEAP Dataset

**Figure 1:**
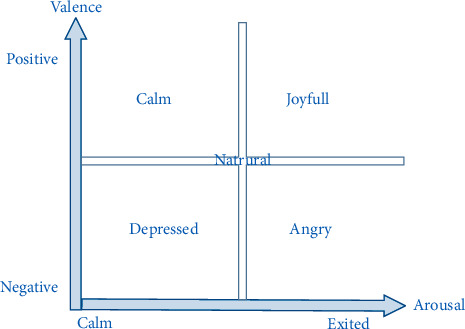
A 2-dimensional arousal-valence model.

The dataset includes brain electrical waves and physiological signals recorded during the user's response to an external stimulus. DEAP is a collection of brain, environmental, and facial signals while watching a music video [26]. In this dataset, 40 music videos have been selected to evoke people's emotions as much as possible. The number of participants in the experiments is 32. Data have been recorded from 40 channels which include 32 EEG channels and 8 physiological channels. The period of each music video is 63 seconds, which includes a 3-second preparation period for watching each music video and one minute for watching. After watching each music video, the participants give a score from 0 to 9 in terms of Valence, Arousal, Dominance, and Liking to each music video. The score that each person gives is considered as a standard criterion for each person. Participants' evaluation of each video is based on the two-dimensional arousal-valence model which is shown in Figure 1 [27].

Arousal: indicates the intensity of people's feelings. The higher the value, the stronger the feeling, and the lower the value, the weaker the feeling. The scale ranges from calm to excited [28].

Valence: indicates the degree of pleasure in the people's feelings. The higher it is, the more positive and happier the person feels, and the lower it is, the more negative and sadder the person feels. The scale ranges from unpleasant to pleasant [28].

The dataset description is given in detail in Table 1. In each of the 32 participant files, the length of data recorded in 63 seconds is 8064 samples, sampled at a frequency rate of 128 Hz. In each of the 32 files, there are physiological and EEG signals recorded from 40 different channels for 40 trials [27].

## 4. Implementation Details

**Figure 2:**
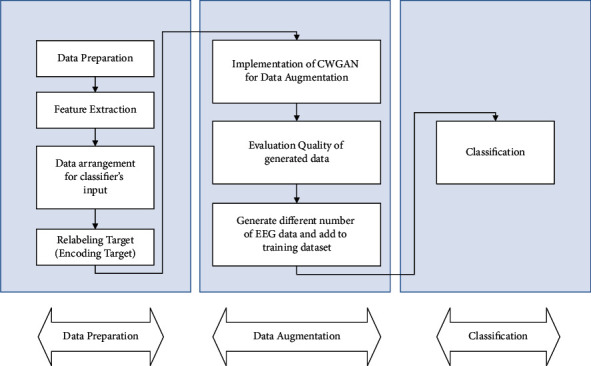
The flowchart of the proposed system.

Traditional feature engineering is one of the oldest solutions for analyzing EEG signals. Depending on the type of problem, features that describe a particular pattern of the signal have been identified and extracted. Feature identification to describe any pattern in brain signals is itself a complex branch of data analysis. According to previous research and their results [22], appropriate features for identifying emotions have been selected and extracted. Feature extraction reduced the dimensions of recorded EEG data. After feature extraction, a data Augmentation method is proposed to generate more data from real EEG data to extend the dataset and overcome the lack of data which leads to overfitting and incorrect prediction of classification models. The general process of recognizing emotions based on traditional feature engineering is shown in Figure 2. Finally, SVM and DNN are used as a classifier to validate the result of augmented data on extracted features.

### 4.1. Data Preparation

**Figure 3:**
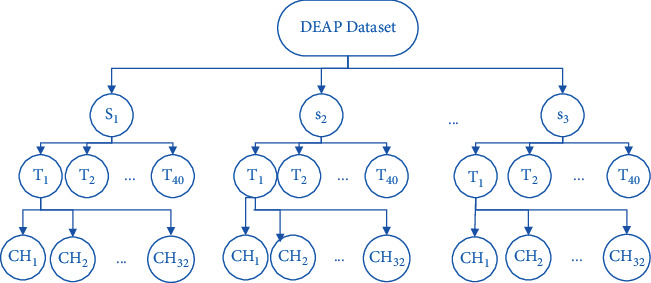
Tree representation of DEAP dataset.

Emotions themselves are a complex issue and relate to many things that are still unknown. Although emotion recognition from EEG signals is an interesting issue, it is too hard to figure out what exactly is going on in a human's mind by analyzing brain activities. Electrical brains might produce different patterns in people's brains in response to the same emotional stimuli. The perplexing EEG dataset is shown in Figure 3.

#### 4.1.1. Data Preparation

**Figure 4:**
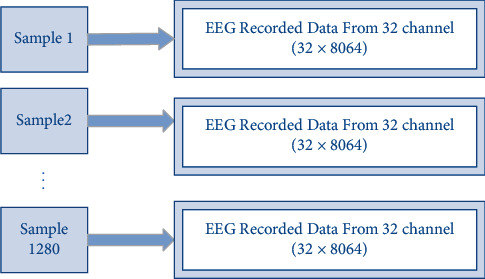
Rearranged DEAP dataset.

As shown in the tree diagram in Figure 3, the recorded data is large and confusing. The first step before solving a problem is a clear definition of the problem. The key point is to clarify what exactly is going to be solved. The first question that arises at first glance is whether we are going to examine and analyze the emotions of one person in different experiments or whether emotions are to be identified between different people. It is important to consider that the emotions of different people in response to the same stimuli may create different emotional patterns in the brain and it is difficult to find a common pattern between them. In this paper, the identification of emotions between different people has been studied and recorded data from all participants in 40 experiments have a total number of 1280 samples. The first preprocessed and rearranged dataset before any exploration is shown in Figure 4.

The dimension of data is still high and takes a long time to be explored and analyzed. Besides, memory usage for this dimension of data is too high. Thus, after rearranging the data, it is time to reduce data dimensionality by doing some feature engineering.

#### 4.1.2. Feature Extraction

In general, feature extraction from EEG signals is one of the most important issues in signal processing. Extracted features from a signal describe the behavior of a signal, and each feature gives special information about data. Therefore, extracting features that can accurately describe signal behavior increases the learning power of machine learning models. If the features extracted from the signal can be easily divided into different classes and the boundary between them is clearer, the machine learning model would be able to learn better. The main purpose of feature extraction is to extract more important information hiding in massive data. Additionally, the feature extraction process also significantly reduces the required resources for data analysis and processing high dimensional data by reducing data processing volume. Time complexity and resource usage is a controversial issue in data analysis and deep neural network-based research. Recently, different techniques have been proposed for feature extraction from EEG signals. So, what is given to a model as input is important. In this paper, many features are extracted from EEG signals as input for machine learning models. The extracted features [22] are shown in Table 2.

All features have been extracted with the help of python libraries and extracted features collocated in a 2d array which is ready to be given to machine learning models. Extracted features describe how brain signals change due to different emotional states. The meaning of each extracted feature is explained accordingly:

- Average PSD: Answers the question “How much of the power of a signal is in a frequency band?”.
- Zero-Crossing Rate (ZCR): It is the number of times the signal changes from positive to negative and vice versa.
- Mean: The value speaks about the mean value of the distribution of data.
- Variance: It shows how the data are spread from the mean of data.

The description of each frequency and what happens in different frequency bands are explained in Table 3.

**Figure 5:**
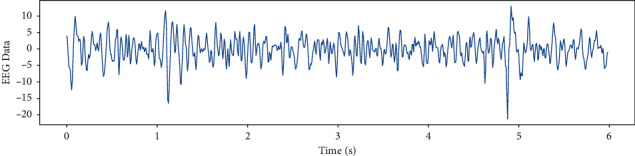
EEG recorded data in 6 (s) from a single channel.

**Figure 6:**
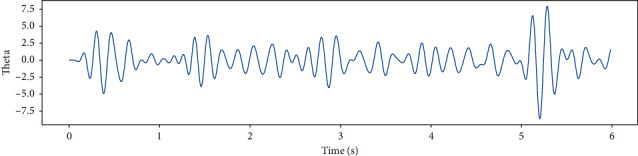
EEG recorded data in the Theta frequency band in 6 (s) from a single channel.

**Figure 7:**
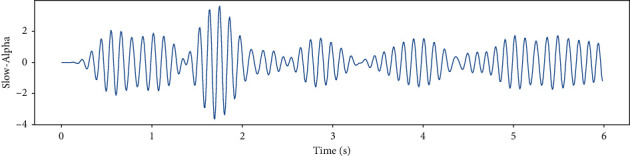
EEG recorded data in the Slow-Alpha frequency band in 6 (s) from a single channel.

**Figure 8:**
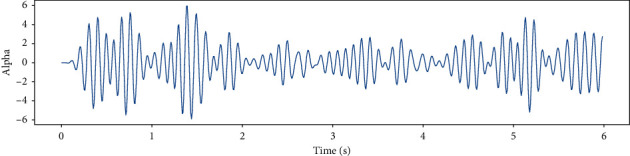
EEG recorded data in the Alpha frequency band in 6 (s) from a single channel.

**Figure 9:**
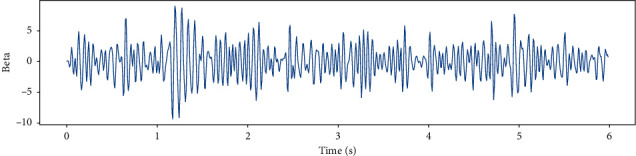
EEG recorded data in the Beta frequency band in 6 (s) from a single channel.

**Figure 10:**
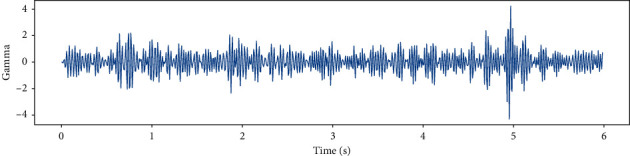
EEG recorded data in Gamma frequency band in 6 (s) from a single channel.

EEG recorded data in 6 (s) from a single channel and EEG data in different frequency bands such as Theta, Slow- Alpha, Alpha, Beta, Gamma are shown in Figures 5–9 and 10.

**Figure 11:**
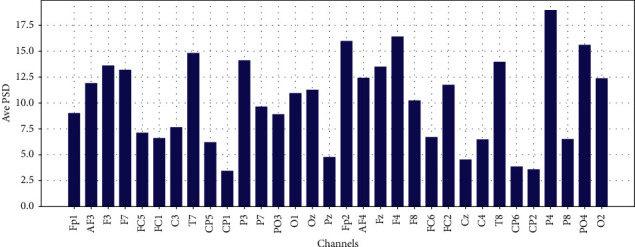
Average PSD for 32 EEG channels in theta frequency band.

**Figure 12:**
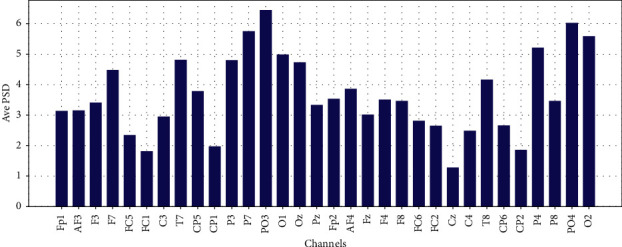
Average PSD for 32 EEG channels in the slow-alpha frequency band.

**Figure 13:**
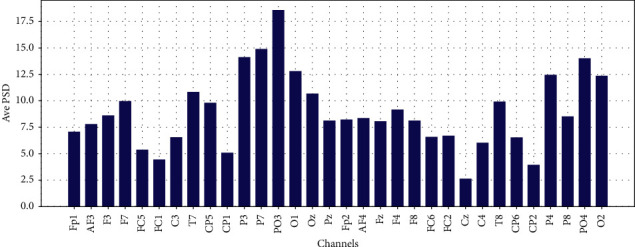
Average PSD for 32 EEG channels in the alpha frequency band.

**Figure 14:**
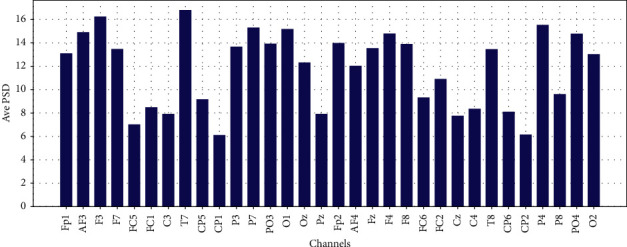
Average PSD for 32 EEG channels in the beta frequency band.

**Figure 15:**
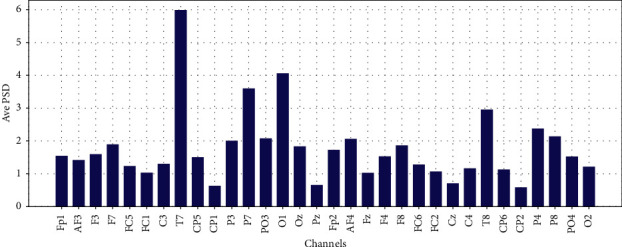
Average PSD for 32 EEG channels in gamma frequency band.

Average PSD in Theta, Low-Alpha, Alpha, Beta, and Gamma for each 32 EEG channels are shown in Figures 11–14 and 15, respectively.

**Figure 16:**
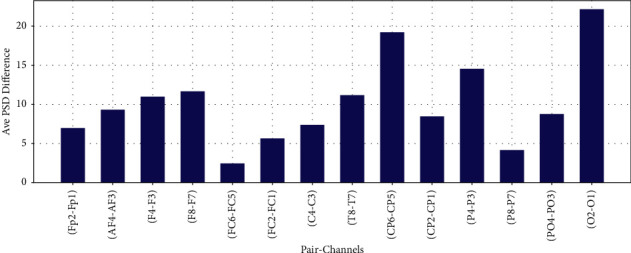
Difference of average PSD in theta frequency band.

**Figure 17:**
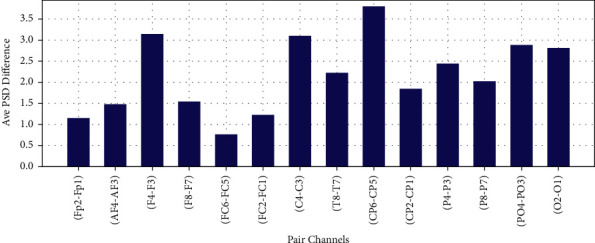
Difference of average PSD in alpha frequency band.

**Figure 18:**
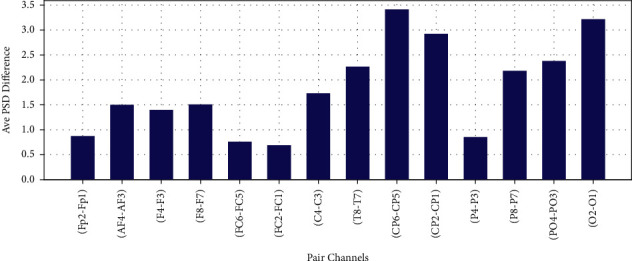
Difference of average PSD in gamma frequency band.

**Figure 19:**
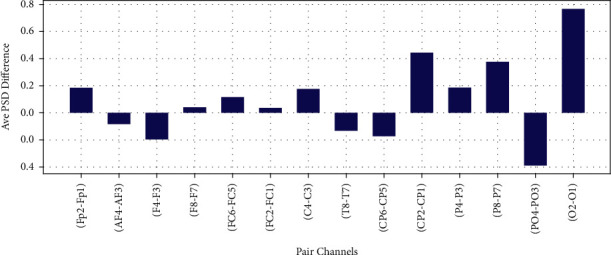
Difference of average PSD in beta frequency band.

The difference of average PSD in Theta, Alpha, Beta, and Gamma bands for 14 EEG channel pairs between right and left scalp are shown in Figures 16–18 and 19, respectively.

**Figure 20:**
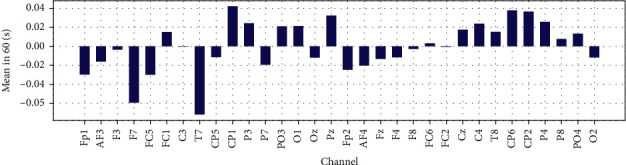
Mean of EEG recorded data for each channel in 60 (s).

**Figure 21:**
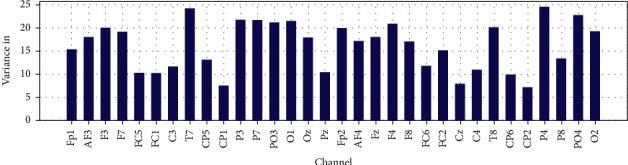
The variance of EEG recorded data for each channel in 60 (s).

**Figure 22:**
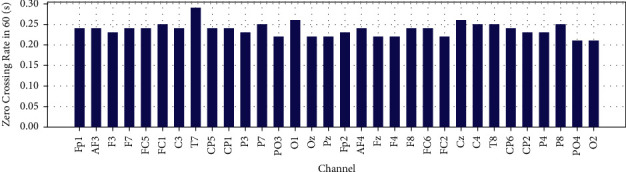
Zero crossing Rate of EEG recorded data for each channel in 60 (s).

Mean, variance and Zero Crossing Rate for 32 EEG channels in 60 are shown in Figures 20, 21, and 22.

#### 4.1.3. Data Arrangement for Classifier's Input

After feature extraction, the extracted features should be arranged in an appropriate format in order to be used as input data of classifiers.

**Figure 23:**
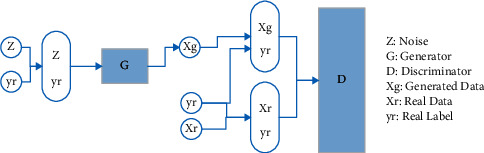
CWGAN network diagram.

*(1) Features matrix*. The EEG features matrix is shown in Figure 23. The rows represent the total number of 32 participants in 40 experiments (1280:32 × 40). The columns represent the extracted features from EEG signals (344).

*(2) Labels matrix*. Based on the scoring value of the participants, the values of the labels ranging from 0–9 are recorded as continuous values. The number 5 has been chosen as the threshold for labeling the upper and lower classes. Hence, scores above 5 are considered as 1, which means high and scores less than or equal to 5 are considered as 0, which means low. Therefore, according to Table 4, the labels are divided into two separate classes, 0 (Low) and 1(High).

*(3) Splitting of Train and Test Data*. To train the proposed model and test if it works properly, the entire available dataset must be divided into two parts: the trainset and the test set. The number of train and test sets is 1152 and 128, respectively.

### 4.2. Data Augmentation

Data augmentation is the process of generating new samples by transforming training data to improve the accuracy and robustness of classifiers [29]. Unfitting methods of increasing data to improve the performance of the model not only do not improve the learning ability of the model but also worsen the result and reduce the predictive power of the model. An appropriate data augmentation method must be chosen based on data properties. Two common data augmentation methods were formerly used in image processing: geometric transformation and noise addition. Geometric transformations, such as shift, scale, rotation/reflection, etc., are not a good choice for augmenting EEG data because it is nonstationary signal and changes over time. The extracted features in the time domain or frequency domain are still time series, so the rotation or shifting of these time series would destroy the features, so it cannot be a suitable technique for this kind of data. Compared with geometric transformation, adding noise is a better choice but not the best method for augmenting EEG data. There are a variety of noises that can be added to data, such as Gaussian, Poisson, Salt, Pepper, etc., but since EEG data is nonstationary, we cannot add any type of these noises to data because it might change the features of EEG data locally. The most frequently used noise for EEG data augmentation based on previous research is Gaussian noise that is added to each feature of the EEG time series to create new data from original data [18]. In our work, we considered using GANs as a very new EEG data augmentation method for generating new data.

### 4.3. GANs

Due to the cost of data collection, most EEG datasets have a small amount of EEG data. Lack of data makes it difficult to predict emotional states with deep learning models that require sufficient training data. In this study, the data enhancement method has been used to solve the lack of data problems in the emotion recognition task. Experimental results have shown that more data can effectively improve the performance of emotion recognition based on deep learning models. Recent work on generative models such as Generative Adversarial Networks (GAN) and Variational Autoencoders (VAEs) have shown that they generate new data like real data. Evidence has also shown artificial data generated by a generative model can be used to increase data, to improve classifier accuracy and prevent overfitting by increasing generalizability [17].

**Figure 24:**
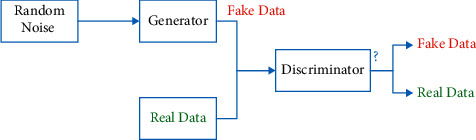
GAN network diagram.

Figure 24 shows how GAN works. Generally, GAN consists of two main components including generator and discriminator that are trying to defeat each other. The input of the generator network is random noise, and the discriminator gets two inputs; generated fake data and real data. It should compare the generated data with real data to recognize whether it is fake or real. The purpose of the generator and discriminator is to fool each other. The generator tries to produce high quality which is like real data to fool discriminator. The discriminator tries to detect fake data. This process continues until the generator produces data that the discriminator cannot recognize whether it is fake or real and consider the generated data as real data. GANs are not able to produce labeled data.

### 4.4. CWGAN Implementation

In [17], the CWGAN network was proposed as a new data augmentation technique to produce EEG data without any judgment about its quality. In this work, not only does the proposed CWGAN produce EEG features, but also the quality of produced data is considered. Therefore, CWGAN is used to generate features that have been previously extracted. Besides, a supplementary condition is considered in generating data to produce labeled data. Then, the quality of produced data is evaluated, and high-quality data is added to the train set. The proposed CWGAN consists of two networks: a generator and a discriminator. These two networks work together to generate realistic-like EEG features. They constantly try to defeat each other. The generator gets a Gaussian noise and a label as input and the discriminator gets two pairs of labeled generated and real data. The generator tries to generate fake data with the same distribution of real data to deceive the discriminator and the discriminator tries to distinguish if the given data is real or fake. The proposed CWGAN works well if the generator can deceive the discriminator. The architecture of CWGAN is shown in Figure 23. The main difference between GAN and CWGAN is that CWGAN produces labeled data.

#### 4.4.1. Generator

**Figure 25:**
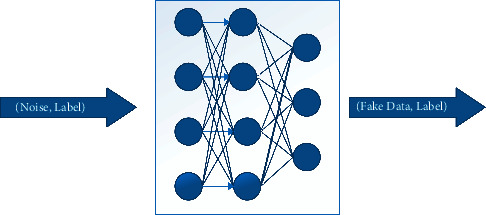
Generator network.

As shown in Figure 25, the generator is designed as a simple deep neural network that gets noise and labels as input and produces fake data from the given noise. Initially, the quality of generated data is adequate. After a few epochs on the generator training process, the generator learns to produce high-quality data to deceive the discriminator. The learning phase is then complete.

#### 4.4.2. Discriminator

**Figure 26:**
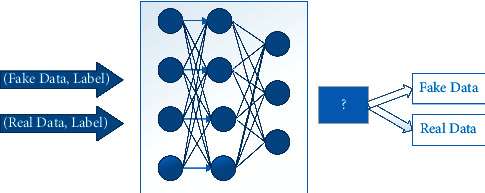
Discriminator network.

It is designed as a simple deep neural network that gets two pairs as input, labeled fake data which is produced by the generator, and labeled real data. The discriminator must distinguish whether the two pairs of given data have the same distribution or not. If it discovers that the distributions of given data are the same, it shows the generator succeeded in deceiving the discriminator by producing high-quality data and the training phase is complete. The discriminator network is shown in Figure 26.

After preparing the training data, the train set is ready to be given as input of the proposed CWGAN to generate more fake data. So, after some preprocessing and normalization on the prepared training set, it is given to the network. Then, by setting the hyper-parameters of CWGAN, it is ready to generate fake data. The quality of the generated data is determined by comparing the distribution diagram of real and generated data and by loss function. The number of training steps is set to 500, and the data generated after 500 steps have high quality. Hyperparameters of the generator and discriminator, i.e., the Epoch number, Batch size, and Learning Rate, are 10, 32, and 0.0002, respectively.

#### 4.4.3. Evaluation Quality of Generated Data

Evaluation of generated high-dimensional EEG data is challenging for researchers. One of the main challenges of using the CWGAN network to generate EEG data is that the quality of generated data cannot be easily identified. Image data can be easily evaluated by visual observation and comparison, but another solution must be sought to evaluate the similarity of produced EEG data with real EEG data. One of the most common methods for comparison is to compare the distribution of generated data with original data. Another technique is to observe the changes in the loss function diagram for the generator and the discriminator during the training phase.

**Figure 27:**
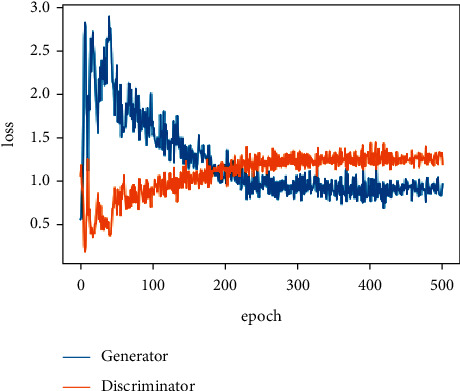
The Loss function of CWGAN networks during training.

**Figure 28:**
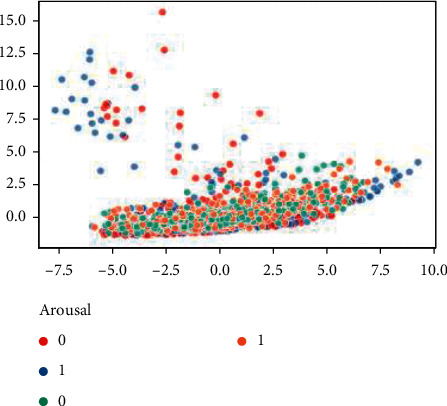
Distribution plot of real and fake data.

Figure 27 describes the changing process of the loss function of the discriminator and generator during training. It shows the process of the CWGAN training phase and the quality of produced data. Initially, the generator begins to generate random data from the noise given to it as input. As shown in Figure 27, the loss of the generator is high, and the loss of the discriminator is low, which means the generator is not able to generate high-quality data and deceive the discriminator. The low loss value for the discriminator means it can distinguish that the given data is fake. The optimum point for high-quality data generation is a low generator loss and a high discriminator loss. When the diagram converges to this point and the changes in loss value become stable, the training phase is complete, and the generated data seems to have a high quality. Also, the distribution of generated and real data must be compared. If they were similar enough, it means that CWGAN was able to generate high-quality data. The distribution of the data is shown in Figure 28, where Z1 and Z2 are the extracted features by PCA with the largest eigenvalues.

Due to the high dimensionality of EEG data, comparing its distribution plot is very difficult. Hence, PCA is applied to generated and real features to reduce the dimension of data for better visualization and comparison. As shown in Figure 18, after 500 training steps, the data generated by CWGAN and its scatter distribution in two-dimensional space are like the real data. Training can be stopped when the scatter plot of generated data becomes equal to the original data and there is little change in the next steps. For image data, its quality can be easily determined by observing and comparing the generated data with original data. Output of this network is ultimately a CSV file that stores a set of generated features and a file that contains the generated labels, which are also formatted as a CSV file.

#### 4.4.4. Adding Generated Data to Train Set

In the next step, generated data is appended to the training set. Various numbers of data have been generated and added to the training set, but only some of them were able to improve the results of classification.

### 4.5. Classification

For data classification, a support vector machine and a deep neural network have been applied to train various sizes of augmented data, and results have shown that in some cases, classification accuracy improved. Contrary to our expectation that increasing data improves classification accuracy, in some cases increasing data not only did not improve accuracy but reduced it.

To implement a stable and efficient deep neural network, a different number of layers and neurons have been tested to reach a high-quality design. Finally, this architecture has yielded the best results. The first layer, which is the input layer, contains 512 neurons, and the hidden layers have 256 and 128 neurons, respectively. After the last hidden layer, a dropout layer is placed to prevent overfitting. The last layer consists of a neuron for binary prediction with the sigmoid activation function, and the middle layers have the Relu activation function.

The network architecture is simple and easy to implement. Low memory consumption and execution time are the issues considered in this research. Support vector machine, which is one of the most powerful machine learning algorithms with easy implementation, high training speed, high predictability, and high stability, is considered for classification. Different kernels have been tested, and it was concluded that the linear kernel was the best in this case.

## 5. Result

For an appropriate training phase, a different number of augmented data and the design of network have been tested. Experimental results are listed in Tables 5 and 6.

As shown in Tables 5 and 6, data augmentation is more efficient in neural network models than standard machine learning models. Data augmentation improved the prediction accuracy of both SVM and DNN classifiers. It was clear that by doubling the data, SVM accuracy improved up to 3.9%, but DNN did not improve at all. The reason is obvious; Deep neural network models require more data than traditional machine learning models. On the one hand, by adding too much data to the original dataset, not only did the accuracy of SVM not improve, but it got worse. On the other hand, adding too much data significantly improved DNN prediction accuracy. DNN prediction accuracy improved up to 6.7%, which is surprisingly noticeable. In conclusion, the data augmentation task, especially in EEG data, is complicated, and so many issues need to be considered. In this experiment, a great number of data have been generated and added to the original dataset, but not all of them effectively yielded the expected result. This means more data does not assuredly improve accuracy. The more important concern in this task is the reliability of the classifier's accuracy. A comparison of the proposed method with previous work is shown in Tables 7 and 8.

## 6. Conclusion

In this study, two challenges were the priority in identifying emotions from EEG signals. The first one is the high dimensionality problem of EEG signals, and the second one lacking EEG data. To solve these problems, feature extraction and data augmentation with generative adversarial networks were, respectively, proposed. The implemented method had a better accuracy on DNN, compared with SVM classifier, which means lack of data is more important for neural network models than traditional machine learning models. The distribution of extracted and generated features has shown that features are heavily cluttered and there is no clear border between the features of different classes. This leads to low classification accuracy, and it is more evident in SVM than DNN.

## 7. Future Work

In this paper, the most important tasks were EEG data generation and feature extraction. The experimental results have shown that extracted features have a key role in the classifier prediction and learning phase. If extracted features have the potential to clearly describe the patterns of signal in different classes, the ability of the classifier increases in prediction and the model works more accurately. Therefore, in future work, feature extraction techniques are considered as the priority of our research criteria. The next problem that leads to the wrong prediction is relabeling data. The binary encoding for the target is one of the reasons of the model's false prediction and low accuracy. For instance, in target encoding, 5.1 is considered as 1, and 4.9 is considered as 0. These two labels are very close to each other and seem to have the same pattern, but they are considered in two different classes of prediction, and it is easy for a model to become confused in prediction, and the false prediction rate increases.

## Figures and Tables

**Table 1:** Dataset description.

Row index	Data	Value
1	Number of participants	32
2	Stimulus	Music video
3	Number of videos	40
4	Duration of each video	60 second
5	EEG recorded data	32 channels
6	Physiological data	8 channels
7	EEG data point for each participant from 32 channel	32 × 8064
8	Labeling method	Done by participants after watching the music video
9	Labeling technique	SAM
10	Label's scale	0 to 9
11	Labels	Arousal, valence, liking, dominance

**Table 2:** Extracted features.

Index	Features type	Channel	Frequency (Hz)	Features	No. of features
1	EEG power features	Fp1, AF3, F3, F7, FC5, FC1, C3, T7, CP5, CP1, P3, P7, PO3, O1, Oz, Pz, Fp2, AF4, Fz, F4, F8, FC6, FC2, Cz, C4, T8, CP6, CP2, P4, P8, PO4, O2	Theta (4–8) slow-alpha (8–10) alpha (8–12) beta (12–30) gamma (30–45)	Average PSD	160

2	EEG power differences	(Fp2- Fp1), (AF4- AF3), (F4-F3), (F8-F7), (FC6-FC5), (FC2-FC1), (C4- C3), (T8-T7), (CP6- CP5), (CP2-CP1), (P4- P3), (P8-P7), (PO4- PO3), (O2- O1)	Theta alpha beta gamma	Difference of average PSD in theta, alpha, beta, and gamma bands for 14 EEG channel pairs between right and left scalp	56

3	EEG time- domain features	Fp1, AF3, F3, F7, FC5, FC1, C3, T7, CP5, CP1, P3, P7, PO3, O1, Oz, Pz, Fp2, AF4, Fz, F4, F8, FC6, FC2, Cz, C4, T8, CP6, CP2, P4, P8, PO4, O2	—	Mean variance zero-crossing rate	128

**Table 3:** EEG Frequency band.

Frequency	Frequency range	Description	Occurrence
Delta 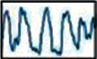	Delta <4/5	Delta frequency band waves have the highest amplitude and the lowest frequency.	It is a wave shape that appears in a relaxed state like deep and unconscious sleep. It describes a person in a state of anesthesia and unconsciousness. Similar EEG frequencies appear in epileptic seizures, loss of consciousness, and some coma states.

Theta 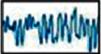	3/5 < theta <7/5	Theta frequency band waves are a fast irregular activity.	Theta waves are associated with natural consciousness or thinking and anxiety and concentration. Beta is usually seen with a symmetrical distribution on both sides of the brain but is more pronounced in the frontal lobe. It may not be present or reduced in areas where the cortex is damaged.

Alpha 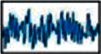	7/5 < alpha <13	Alpha frequency band waves are generated by the simultaneous electrical activity of large groups of neurons.	They are usually found with the eyes closed but still awake in signals recorded from the scalp more than the occipital lobe during periods of relaxation. Open eyes also reduce drowsiness and sleepiness. It mostly indicates a state of consciousness

Beta 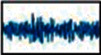	12 < beta <25	Beta frequency band waves are a fast irregular activity, where the cortex is damaged.	Beta waves are associated with natural consciousness or thinking and anxiety and concentration. Beta usually occurs on both sides of the brain with a symmetrical distribution but is mainly seen in the frontal lobe. It may not be present or reduced in areas

Gamma 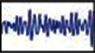	26 < gamma <70	Gamma waves are thought to be a sign of the active exchange of information between the cerebral cortex and other areas.	Gamma waves are usually generated in the brain when people are conscious and when the eyes move rapidly. Gamma and beta waves may overlap within the range of natural frequencies, and the exact boundary between these two frequency bands is not clear and yet is a judgment for experts.

**Table 4:** Encoded target.

Row_ Index	Arousal	Valence
**1**	**0**	**1**
**2**	**1**	**1**
**3**	**1**	**0**
**…**	**…**	**…**
**1280**	**0**	**1**

**Table 5:** SVM classification results.

Augmented data real data + fake data	NO. test data	Arousal mean accuracy STD (%)		Valence mean accuracy STD (%)	
1152 + 0	128	64.3	% ± 2.3	60.1	% ± 5.2
**1152** **+** **1152**	**128**	**68.2**	**%**±**4.7**	**64.3**	**%**±**4.8**
1152 + 5000	128	62.4	% ± 3.8	59.6	% ± 3.1

**Table 6:** DNN classification results.

Augmented data real data + fake data	NO. test data	Arousal mean accuracy STD (%)		Valence mean accuracy STD (%)	
1152 + 0	128	65.4	% ± 3.4	64.3	% ± 4.3
1152 + 1152	128	65.2	% ± 4.2	62.3	% ± 4.7
**1152** **+** **5000**	**128**	**71.9**	**%**±**4.8**	**67.4**	**%**±**5.2**

**Table 7:** Comparison of proposed work with similar work on SVM classifier.

Model	Features	NO. Augmented data	Classification type	Mean accuracy	STD	Arousal		Valence	
						M.Acc	STD	M.Acc	STD
Proposed model	344 extracted features	0	Binary	—	—	64.3%	% ± 2.3	60.1%	% ± 5.2
Proposed model	344 extracted features	2 × real data	Binary	—	—	**68.2%**	**%**±**4.7**	**64.3%**	**%**±**4.8**
Proposed model	344 extracted features	Real data + 5000	Binary	—	—	62.4%	% ± 3.8	59.6%	% ± 3.1
[1]	DE	0	Categorical	45.4%	8.2%	—	—	—	—
[1]	DE	**5000**	Categorical	**48.9%**	**8.4%**	—	—	—	—
[1]	PSD	0	Categorical	42.7%	9.6%	—	—	—	—
[1]	PSD	5000	Categorical	45.0%	8.9%	—	—	—	—

**Table 8:** Comparison of proposed work with similar work on DNN classifier.

Model	Features	NO. augmented data	Classification type	Mean accuracy	STD	Arousal		Valence	
						M.Acc	STD	M.Acc	STD
Proposed model	344 extracted features	0	Binary	—	—	65.4%	% ± 3.4	64.3%	% ± 4.3
Proposed model	344 extracted features	2 × real data	Binary	—	—	65.2%	% ± 4.2	62.3%	% ± 4.7
Proposed model	344 extracted features	Real data + 5000	Binary	—	—	**71.9%**	**%**±**4.8**	**67.4%**	**%**±**5.2**
[1]	DE	0	Categorical	44.9%	4.0%	—	—	—	—
[1]	DE	**5000**	Categorical	**46.9%**	**4.8%**	—	—	—	—

## Data Availability

To gain access to the dataset and download the files, please visit links below in order to obtain a username and a password: https://www.eecs.qmul.ac.uk/mmv/datasets/deap/download.htmlhttps://anaxagoras.eecs.qmul.ac.uk/request.php?dataset=DEAP.
